# Supplementary material for: P-TRAP: a Panicle Trait Phenotyping tool
Source: BMC Plant Biol. 2013 Aug 29;13:122. doi: 10.1186/1471-2229-13-122 (PMC3848748; doi:10.1186/1471-2229-13-122)
Supplement: Additional file 7 — P-TRAP source code. The Netbeans project that contains the source code of the application. [file 1471-2229-13-122-S7.zip › javadoc.zip!/javadoc/org-basmati-workspace/org/basmati/workspace/GraphUI.html]

GraphUI (WorkSpace)


JavaScript is disabled on your browser.


- Package
- Class
- Tree
- Deprecated
- Index
- Help

*org.basmati.workspace 1.0*

- Prev Class
- Next Class

- Frames
- No Frames

- All Classes

- Summary:
- Nested |
- Field |
- Constr |
- Method

- Detail:
- Field |
- Constr |
- Method


org.basmati.workspace

## Class GraphUI

- java.lang.Object
- - org.netbeans.api.visual.widget.Widget
  - - org.netbeans.api.visual.widget.Scene
    - - org.netbeans.api.visual.model.ObjectScene
      - - org.netbeans.api.visual.graph.GraphScene<java.lang.String,java.lang.String>
        - - org.basmati.workspace.GraphUI

- All Implemented Interfaces:
  :   javax.accessibility.Accessible, org.openide.util.Lookup.Provider

  ---

    

  ```
  public class GraphUI
  extends org.netbeans.api.visual.graph.GraphScene<java.lang.String,java.lang.String>
  ```

  The GraphScene which shows the structure as a graph.

  Author:
  :   faroq

- - ### Nested Class Summary

    Nested Classes

    | Modifier and Type | Class and Description |
    | --- | --- |
    | `static class` | `GraphUI.calcType` This is the type of the calculation required on the graph |

    - ### Nested classes/interfaces inherited from class org.netbeans.api.visual.graph.GraphScene

      `org.netbeans.api.visual.graph.GraphScene.StringGraph`
    - ### Nested classes/interfaces inherited from class org.netbeans.api.visual.widget.Scene

      `org.netbeans.api.visual.widget.Scene.SceneListener`
    - ### Nested classes/interfaces inherited from class org.netbeans.api.visual.widget.Widget

      `org.netbeans.api.visual.widget.Widget.Dependency`
  - ### Constructor Summary

    Constructors

    Constructor and Description

    `GraphUI()`
  - ### Method Summary

    Methods

    | Modifier and Type | Method and Description |
    | --- | --- |
    | `protected void` | `attachEdgeSourceAnchor(java.lang.String e, java.lang.String oldsourceNode, java.lang.String sourceNode)` |
    | `protected void` | `attachEdgeTargetAnchor(java.lang.String e, java.lang.String oldtargetNode, java.lang.String targetNode)` |
    | `protected org.netbeans.api.visual.widget.Widget` | `attachEdgeWidget(java.lang.String e)` |
    | `protected org.netbeans.api.visual.widget.Widget` | `attachNodeWidget(java.lang.String point)` |
    | `void` | `calcpb()` Calculate the main level branches |
    | `void` | `calcsb()` Calculate the second level branches |
    | `void` | `calctb()` Calculate the third level branches |
    | `void` | `clear()` |
    | `int` | `countMainGeneratingPoints(java.util.ArrayList<org.bioalg.widgetfactory.CircleWidget> circleWidgets)` |
    | `void` | `DrawGraphModel()` This function takes a Graphmodel and convert it to an editable graph on the scene |
    | `void` | `fit2view(javax.swing.JScrollPane scrollpane)` |
    | `org.bioalg.widgetfactory.CircleWidget` | `getbestNeighbor(org.bioalg.widgetfactory.CircleWidget circleWidget, GraphUI.calcType type)` In graph decomposition this function searches for the "winner" vertex to be followed during the decomposition. |
    | `org.bioalg.graphprocessor.GraphModel` | `getGm()` |
    | `double` | `getGravity()` |
    | `org.openide.filesystems.FileObject` | `getImagefile()` |
    | `int` | `getleftmost(java.util.ArrayList<org.bioalg.widgetfactory.CircleWidget> startend)` |
    | `double` | `getMeanBranchesLength()` |
    | `java.util.ArrayList<org.bioalg.widgetfactory.CircleWidget>` | `getNeighbors(org.bioalg.widgetfactory.CircleWidget widget)` A helper function to get the neighbors of a given widget |
    | `java.util.ArrayList<org.bioalg.widgetfactory.CircleWidget>` | `getneighborsofColor(java.util.ArrayList<org.bioalg.widgetfactory.CircleWidget> neighbors, java.awt.Color x)` |
    | `java.util.ArrayList<org.bioalg.widgetfactory.CircleWidget>` | `getneighborsofColor(java.util.ArrayList<org.bioalg.widgetfactory.CircleWidget> neighbors, java.awt.Color x, org.bioalg.widgetfactory.CircleWidget exculde)` |
    | `java.util.ArrayList<org.bioalg.widgetfactory.CircleWidget>` | `getneighborsofColor(java.util.ArrayList<org.bioalg.widgetfactory.CircleWidget> neighbors, java.awt.Color x1, java.awt.Color x2)` |
    | `java.util.ArrayList<org.bioalg.widgetfactory.CircleWidget>` | `getneighborsofColor(java.util.ArrayList<org.bioalg.widgetfactory.CircleWidget> neighbors, java.awt.Color x1, java.awt.Color x2, java.awt.Color x3, org.bioalg.widgetfactory.CircleWidget cir)` |
    | `int` | `getrightmost(java.util.ArrayList<org.bioalg.widgetfactory.CircleWidget> startend)` |
    | `java.util.ArrayList<org.bioalg.widgetfactory.CircleWidget>` | `getStartandEndPoints()` Get start and end points |
    | `void` | `getVertexGravity(org.bioalg.widgetfactory.CircleWidget circleWidget, GraphUI.calcType type)` Calculate the weight of the vertex. |
    | `void` | `highlightPaths(org.bioalg.graphprocessor.Vertex vertex)` **Deprecated.** |
    | `void` | `increamental()` |
    | `boolean` | `isallwithColorX(java.util.ArrayList<org.bioalg.widgetfactory.CircleWidget> circleWidgets, java.awt.Color color)` |
    | `boolean` | `isDirty()` |
    | `boolean` | `isMainJunction(org.bioalg.widgetfactory.CircleWidget widget, java.util.ArrayList<org.bioalg.widgetfactory.CircleWidget> circleWidgets)` |
    | `org.bioalg.graphprocessor.Vertex.VERTEX_TYPE` | `mapColor2Type(java.awt.Color c)` Maps the input color to a vertex type. |
    | `java.awt.Color` | `mapType2Color(org.bioalg.graphprocessor.Vertex.VERTEX_TYPE c)` Maps the input vertex type to a color |
    | `boolean` | `markTransitions()` Detect if there are any transition vertices in the graph. |
    | `double` | `measureLength()` **Deprecated.** *Now the user define the scale in the source image at the `ImageViewScene` class.* |
    | `int` | `neighborsOfNumberX(java.util.ArrayList<org.bioalg.widgetfactory.CircleWidget> neighbors, java.awt.Color x)` |
    | `int` | `neighborsOfNumberX(java.util.ArrayList<org.bioalg.widgetfactory.CircleWidget> neighbors, java.awt.Color c1, java.awt.Color c2)` |
    | `void` | `reset()` |
    | `void` | `restallLabels()` |
    | `void` | `setcolor(java.util.ArrayList<org.bioalg.widgetfactory.CircleWidget> widgets, java.awt.Color c)` |
    | `void` | `setDirty(boolean dirty)` |
    | `void` | `setGm(org.bioalg.graphprocessor.GraphModel gm)` |
    | `void` | `setImagefile(org.openide.filesystems.FileObject imagefile)` |
    | `void` | `setupLayers(java.awt.Image image)` |
    | `void` | `setupLogicalModels(org.bioalg.graphprocessor.GraphModel gm, org.openide.filesystems.FileObject imagefile)` |
    | `void` | `updateGraphModel()` Update the graph model when user have made some changes. |

    - ### Methods inherited from class org.netbeans.api.visual.graph.GraphScene

      `addEdge, addNode, detachEdgeWidget, detachNodeWidget, findEdgesBetween, findNodeEdges, getEdges, getEdgeSource, getEdgeTarget, getNodes, isEdge, isNode, notifyEdgeAdded, notifyNodeAdded, removeEdge, removeNode, removeNodeWithEdges, setEdgeSource, setEdgeTarget`
    - ### Methods inherited from class org.netbeans.api.visual.model.ObjectScene

      `addObject, addObjectSceneListener, createObjectHoverAction, createSelectAction, findObject, findStoredObject, findWidget, findWidgets, getFocusedObject, getHighlightedObjects, getHoveredObject, getIdentityCode, getObjects, getObjectState, getSelectedObjects, isObject, removeObject, removeObjectSceneListener, setFocusedObject, setHighlightedObjects, setHoveredObject, setSelectedObjects, userSelectionSuggested`
    - ### Methods inherited from class org.netbeans.api.visual.widget.Scene

      `addSceneListener, convertSceneToView, convertSceneToView, convertViewToScene, createBirdView, createSatelliteView, createView, createWidgetHoverAction, getActiveTool, getDefaultFont, getFocusedWidget, getGraphics, getInputBindings, getKeyEventProcessingType, getLookFeel, getMaximumBounds, getPriorActions, getResourceTable, getSceneAnimator, getView, getZoomFactor, isRepaintRequiredForRevalidating, isValidated, paint, removeSceneListener, setActiveTool, setFocusedWidget, setKeyEventProcessingType, setLookFeel, setMaximumBounds, setResourceTable, setZoomFactor, validate, validate`
    - ### Methods inherited from class org.netbeans.api.visual.widget.Widget

      `addChild, addChild, addChild, addChild, addChildren, addDependency, bringToBack, bringToFront, calculateClientArea, convertLocalToScene, convertLocalToScene, convertSceneToLocal, convertSceneToLocal, createActions, equals, getAccessibleContext, getActions, getActions, getBackground, getBorder, getBounds, getChildConstraint, getChildren, getClientArea, getCursor, getCursorAt, getDependencies, getFont, getForeground, getLayout, getLocation, getLookup, getMaximumSize, getMinimumSize, getParentWidget, getPreferredBounds, getPreferredLocation, getPreferredSize, getScene, getState, getToolTipText, hashCode, isCheckClipping, isEnabled, isHitAt, isOpaque, isPreferredBoundsSet, isVisible, notifyAdded, notifyBackgroundChanged, notifyFontChanged, notifyForegroundChanged, notifyRemoved, notifyStateChanged, paint, paintBackground, paintBorder, paintChildren, paintWidget, removeChild, removeChildren, removeChildren, removeDependency, removeFromParent, repaint, resolveBounds, revalidate, revalidate, setAccessibleContext, setBackground, setBackgroundFromResource, setBorder, setBorder, setCheckClipping, setChildConstraint, setCursor, setEnabled, setFont, setFontFromResource, setForeground, setForegroundFromResource, setLayout, setMaximumSize, setMinimumSize, setOpaque, setPreferredBounds, setPreferredLocation, setPreferredSize, setState, setToolTipText, setVisible, updateResources`
    - ### Methods inherited from class java.lang.Object

      `clone, finalize, getClass, notify, notifyAll, toString, wait, wait, wait`

- - ### Constructor Detail


    - #### GraphUI

      ```
      public GraphUI()
      ```
  - ### Method Detail


    - #### isDirty

      ```
      public boolean isDirty()
      ```


    - #### setDirty

      ```
      public void setDirty(boolean dirty)
      ```


    - #### setupLayers

      ```
      public void setupLayers(java.awt.Image image)
      ```


    - #### setupLogicalModels

      ```
      public void setupLogicalModels(org.bioalg.graphprocessor.GraphModel gm,
                            org.openide.filesystems.FileObject imagefile)
      ```


    - #### getGm

      ```
      public org.bioalg.graphprocessor.GraphModel getGm()
      ```


    - #### setGm

      ```
      public void setGm(org.bioalg.graphprocessor.GraphModel gm)
      ```


    - #### getImagefile

      ```
      public org.openide.filesystems.FileObject getImagefile()
      ```


    - #### setImagefile

      ```
      public void setImagefile(org.openide.filesystems.FileObject imagefile)
      ```


    - #### DrawGraphModel

      ```
      public void DrawGraphModel()
      ```

      This function takes a Graphmodel and convert it to an editable graph on the scene


    - #### attachNodeWidget

      ```
      protected org.netbeans.api.visual.widget.Widget attachNodeWidget(java.lang.String point)
      ```

      **Specified by:**
      :   `attachNodeWidget` in class `org.netbeans.api.visual.graph.GraphScene<java.lang.String,java.lang.String>`


    - #### attachEdgeWidget

      ```
      protected org.netbeans.api.visual.widget.Widget attachEdgeWidget(java.lang.String e)
      ```

      **Specified by:**
      :   `attachEdgeWidget` in class `org.netbeans.api.visual.graph.GraphScene<java.lang.String,java.lang.String>`


    - #### attachEdgeSourceAnchor

      ```
      protected void attachEdgeSourceAnchor(java.lang.String e,
                                java.lang.String oldsourceNode,
                                java.lang.String sourceNode)
      ```

      **Specified by:**
      :   `attachEdgeSourceAnchor` in class `org.netbeans.api.visual.graph.GraphScene<java.lang.String,java.lang.String>`


    - #### attachEdgeTargetAnchor

      ```
      protected void attachEdgeTargetAnchor(java.lang.String e,
                                java.lang.String oldtargetNode,
                                java.lang.String targetNode)
      ```

      **Specified by:**
      :   `attachEdgeTargetAnchor` in class `org.netbeans.api.visual.graph.GraphScene<java.lang.String,java.lang.String>`


    - #### getGravity

      ```
      public double getGravity()
      ```


    - #### getVertexGravity

      ```
      public void getVertexGravity(org.bioalg.widgetfactory.CircleWidget circleWidget,
                          GraphUI.calcType type)
      ```

      Calculate the weight of the vertex.

      Parameters:
      :   `circleWidget` - `CircleWidget`
      :   `type` - `GraphUI.calcType`


    - #### getbestNeighbor

      ```
      public org.bioalg.widgetfactory.CircleWidget getbestNeighbor(org.bioalg.widgetfactory.CircleWidget circleWidget,
                                                          GraphUI.calcType type)
      ```

      In graph decomposition this function searches for the "winner" vertex to be followed during the decomposition.

      Parameters:
      :   `circleWidget` -
      :   `type` -

      Returns:


    - #### getMeanBranchesLength

      ```
      public double getMeanBranchesLength()
      ```


    - #### countMainGeneratingPoints

      ```
      public int countMainGeneratingPoints(java.util.ArrayList<org.bioalg.widgetfactory.CircleWidget> circleWidgets)
      ```


    - #### getleftmost

      ```
      public int getleftmost(java.util.ArrayList<org.bioalg.widgetfactory.CircleWidget> startend)
      ```


    - #### getrightmost

      ```
      public int getrightmost(java.util.ArrayList<org.bioalg.widgetfactory.CircleWidget> startend)
      ```


    - #### calcpb

      ```
      public void calcpb()
      ```

      Calculate the main level branches


    - #### calctb

      ```
      public void calctb()
      ```

      Calculate the third level branches


    - #### setcolor

      ```
      public void setcolor(java.util.ArrayList<org.bioalg.widgetfactory.CircleWidget> widgets,
                  java.awt.Color c)
      ```


    - #### calcsb

      ```
      public void calcsb()
      ```

      Calculate the second level branches


    - #### increamental

      ```
      public void increamental()
      ```


    - #### reset

      ```
      public void reset()
      ```


    - #### markTransitions

      ```
      public boolean markTransitions()
      ```

      Detect if there are any transition vertices in the graph. This is what shows the yellow "?" sign when an error detected.

      Returns:
      :   true if detected some, false otherwise.


    - #### getStartandEndPoints

      ```
      public java.util.ArrayList<org.bioalg.widgetfactory.CircleWidget> getStartandEndPoints()
      ```

      Get start and end points

      Returns:


    - #### neighborsOfNumberX

      ```
      public int neighborsOfNumberX(java.util.ArrayList<org.bioalg.widgetfactory.CircleWidget> neighbors,
                           java.awt.Color x)
      ```


    - #### getneighborsofColor

      ```
      public java.util.ArrayList<org.bioalg.widgetfactory.CircleWidget> getneighborsofColor(java.util.ArrayList<org.bioalg.widgetfactory.CircleWidget> neighbors,
                                                                                   java.awt.Color x)
      ```


    - #### getneighborsofColor

      ```
      public java.util.ArrayList<org.bioalg.widgetfactory.CircleWidget> getneighborsofColor(java.util.ArrayList<org.bioalg.widgetfactory.CircleWidget> neighbors,
                                                                                   java.awt.Color x1,
                                                                                   java.awt.Color x2)
      ```


    - #### getneighborsofColor

      ```
      public java.util.ArrayList<org.bioalg.widgetfactory.CircleWidget> getneighborsofColor(java.util.ArrayList<org.bioalg.widgetfactory.CircleWidget> neighbors,
                                                                                   java.awt.Color x1,
                                                                                   java.awt.Color x2,
                                                                                   java.awt.Color x3,
                                                                                   org.bioalg.widgetfactory.CircleWidget cir)
      ```


    - #### getneighborsofColor

      ```
      public java.util.ArrayList<org.bioalg.widgetfactory.CircleWidget> getneighborsofColor(java.util.ArrayList<org.bioalg.widgetfactory.CircleWidget> neighbors,
                                                                                   java.awt.Color x,
                                                                                   org.bioalg.widgetfactory.CircleWidget exculde)
      ```


    - #### neighborsOfNumberX

      ```
      public int neighborsOfNumberX(java.util.ArrayList<org.bioalg.widgetfactory.CircleWidget> neighbors,
                           java.awt.Color c1,
                           java.awt.Color c2)
      ```


    - #### isallwithColorX

      ```
      public boolean isallwithColorX(java.util.ArrayList<org.bioalg.widgetfactory.CircleWidget> circleWidgets,
                            java.awt.Color color)
      ```


    - #### isMainJunction

      ```
      public boolean isMainJunction(org.bioalg.widgetfactory.CircleWidget widget,
                           java.util.ArrayList<org.bioalg.widgetfactory.CircleWidget> circleWidgets)
      ```


    - #### getNeighbors

      ```
      public java.util.ArrayList<org.bioalg.widgetfactory.CircleWidget> getNeighbors(org.bioalg.widgetfactory.CircleWidget widget)
      ```

      A helper function to get the neighbors of a given widget

      Parameters:
      :   `widget` - A `CircleWidget` object.

      Returns:
      :   an arraylist object of widgets that contains the node neighbors


    - #### updateGraphModel

      ```
      public void updateGraphModel()
      ```

      Update the graph model when user have made some changes.


    - #### mapColor2Type

      ```
      public org.bioalg.graphprocessor.Vertex.VERTEX_TYPE mapColor2Type(java.awt.Color c)
      ```

      Maps the input color to a vertex type.

      Parameters:
      :   `c` - the input color

      Returns:
      :   a vertex type

      See Also:
      :   `Vertex`,
          `Color`


    - #### mapType2Color

      ```
      public java.awt.Color mapType2Color(org.bioalg.graphprocessor.Vertex.VERTEX_TYPE c)
      ```

      Maps the input vertex type to a color

      Parameters:
      :   `c` - a vertex type.

      Returns:
      :   A color object

      See Also:
      :   `Vertex`,
          `Color`


    - #### highlightPaths

      ```
      public void highlightPaths(org.bioalg.graphprocessor.Vertex vertex)
      ```

      Deprecated.

      Used to highlight the path by using the mouse.

      Parameters:
      :   `vertex` - the hovered generated vertex


    - #### restallLabels

      ```
      public void restallLabels()
      ```


    - #### measureLength

      ```
      public double measureLength()
      ```

      Deprecated. *Now the user define the scale in the source image at the `ImageViewScene` class.*

      Measure length between two widgets, only two widgets are expected, this
      is used to define a measuring unit.

      Returns:
      :   a double value that represent the length


    - #### clear

      ```
      public void clear()
      ```


    - #### fit2view

      ```
      public void fit2view(javax.swing.JScrollPane scrollpane)
      ```


- Package
- Class
- Tree
- Deprecated
- Index
- Help

*org.basmati.workspace 1.0*

- Prev Class
- Next Class

- Frames
- No Frames

- All Classes

- Summary:
- Nested |
- Field |
- Constr |
- Method

- Detail:
- Field |
- Constr |
- Method
